# Effects of immediate and delayed loading protocols on marginal bone loss around implants in unsplinted mandibular implant-retained overdentures: a systematic review and meta-analysis

**DOI:** 10.1186/s12903-021-01486-3

**Published:** 2021-03-17

**Authors:** Wei Liu, He Cai, Junjiang Zhang, Jian Wang, Lei Sui

**Affiliations:** 1 Department of Prosthodontics, School & Hospital of Stomatology, Tianjin Medical University, Tianjin, 300070 China; 2 State Key Laboratory of Oral Diseases, National Clinical Research Center for Oral Diseases, West China School of Stomatology, Sichuan University, No. 14, Section 3, South Renmin Road, Chengdu, 610041 China; 3 Department of Prosthodontics, West China Hospital of Stomatology, Sichuan University, No. 14, Section 3, South Renmin Road, Chengdu, 610041 China

**Keywords:** Implant-retained overdenture, Immediate loading, Delayed loading, Marginal bone loss, Systematic review, Meta-analysis

## Abstract

**Background:**

Immediate loading has recently been introduced into unsplinted mandibular implant-retained overdentures for the management of edentulous patients due to their increasing demand on immediate aesthetics and function. However, there is still a scarcity of meta-analytical evidence on the efficacy of immediate loading compared to delayed loading in unsplinted mandibular implant-retained overdentures. The purpose of this study was to compare the marginal bone loss (MBL) around implants between immediate and delayed loading of unsplinted mandibular implant-retained overdentures.

**Methods:**

Randomized controlled trials (RCTs), controlled clinical trials (CCTs), and cohort studies quantitatively comparing the MBL around implants between immediate loading protocol (ILP) and delayed loading protocol (DLP) of unsplinted mandibular overdentures were included. A systematic search was carried out in PubMed, EMBASE, and CENTRAL databases on December 02, 2020. “Grey” literature was also searched. A meta-analysis was conducted to compare the pooled MBL of two different loading protocols of unsplinted mandibular overdentures through weighted mean differences (WMDs) with 95% confidence intervals (95% CIs). The subgroup analysis was performed between different attachment types (i.e. Locator attachment vs. ball anchor). The risk of bias within and across studies were assessed using the Cochrane Collaboration’s tool, the Newcastle–Ottawa scale, and Egger’s test.

**Results:**

Of 328 records, five RCTs and two cohort studies were included and evaluated, which totally contained 191 participants with 400 implants. The MBL of ILP group showed no significant difference with that of DLP group (WMD 0.04, CI − 0.13 to 0.21, *P* > .05). The subgroup analysis revealed similar results with Locator attachments or ball anchors (*P* > .05). Apart from one RCT (20%) with a high risk of bias, four RCTs (80%) showed a moderate risk of bias. Two prospective cohort studies were proved with acceptable quality. Seven included studies have reported 5.03% implant failure rate (10 of 199 implants) in ILP group and 1.00% failure rate (2 of 201 implants) in DLP group in total.

**Conclusions:**

For unsplinted mandibular implant-retained overdentures, the MBL around implants after ILP seems comparable to that of implants after DLP. Immediate loading may be a promising alternative to delayed loading for the management of unsplinted mandibular implant-retained overdentures.

**PROSPERO registration number**: CRD42020159124.

**Supplementary Information:**

The online version contains supplementary material available at 10.1186/s12903-021-01486-3.

## Background

The rehabilitation of edentulism has long been regarded as one of the main challenges for dentists. Conventional complete dentures may present some limitations such as insufficient retention and poor comfort, especially in the severely atrophic mandible [1]. With the wide application of osseointegrated implants, implant-retained overdentures have been introduced as a viable alternative to conventional complete dentures [2, 3]. In order to obtain adequate retention at a reasonable cost, the McGill Consensus Statement [4] recommended that the overdenture retained by two implants in anterior area of mandible could be the first-choice standard for edentulous patients. More recently, the York Consensus Statement [5] provided further clinical evidence that the use of no less than two implants could significantly enhance the retention of mandibular overdentures and the EAO consensus conference [6] also indicated that the oral health-related of life outcomes could be improved with overdentures retained by two or four implants. Little difference was observed between the average annual costs of overdentures retained by two implants and conventional complete dentures. Moreover, when unsplinted anchors are employed, the implant-retained overdentures could cost even less compared to their counterparts with splinted anchors [5]. Besides low costs, the unsplinted mandibular implant-retained overdentures display a low repair frequency and easy maintenance on isolated attachments, attracting more attention in the clinic [7]. Furthermore, the unsplinted overdentures provide good emergence profile and require simple oral hygiene practice in daily life [8]. Accordingly, unsplinted mandibular implant-retained overdentures with at least two implants have been frequently used in dental clinic [9–11].

Long-term outcomes of implant-retained overdentures are greatly affected by the longevity and functionality of the underlying implants, and the osseointegration is considered as the most important determinant of implant success [12]. To achieve osseointegration, Branemark et al. [13] suggested that sufficient healing time after implant insertion should be provided. Accordingly, a delayed loading protocol (DLP) was routinely performed, in which the prosthesis would be attached to the implants in the second stage after a healing period of three to six months [14, 15]. For a long time, it was assumed that enough healing time of DLP could not only decrease the risk of infection but also avoid the micromotion at the bone-implant interface that might lead to the implant failure [13, 16, 17]. However, along with lots of researches on intraosseous implant materials and implant surface modification techniques during the last two decades, predictable osseointegration could be achieved with obviously reduced healing time [18]. More importantly, optimized implant geometry designs and improved surgical techniques significantly enhanced the initial stability of dental implants [19, 20]. Therefore, the necessity of long healing period was challenged and the change of loading protocol was required to address the increasing demands of patients on the immediate aesthetics and function [21, 22]. Consequently, immediate loading protocol (ILP), in which the implants could be put in function within seven days after their placement [23], has been introduced into clinical practice [24]. But the prognosis of implants restored with ILP is yet to be further evaluated, especially regarding the promising unsplinted mandibular implant-retained overdentures.

Marginal bone loss (MBL), measured as the bone loss from the implant neck to the first bone-to-implant contact [25], is recognized as a crucial consideration for the attainment and maintenance of implant osseointegration [26]. In general, remodelling and resorption of peri-implant marginal bone could be observed after the implant surgery without bone augmentation technique. Therefore, MBL in the first year of loading was proposed as a criterion for implant success [27–29] and many studies have focused on the threshold between normal and pathological MBL. Papaspyridakos et al. [30] suggested that MBL of less than 1.5 mm in the first year was one of evaluation criteria for clinical implant success. Roos-Jansaker et al. [28] reported that MBL of less than 1.8 mm presented a reliable clinical outcome, while Naert et al. [31] believed that the maximal mean bone loss of 2.2 mm still indicated an acceptable long-term outcome. In spite of some discrepancies, MBL of 2 mm is wildly accepted as the maximum threshold of MBL in clinic situations, and is frequently used to evaluate the implant success as well as the extent of peri-implantitis [32, 33].

Several systematic reviews have focused on the effects of these two different loading protocols on MBL around implants in fixed and removable restorations [34, 35], while there was no meta-analytic review regarding the comparison between ILP and DLP in unsplinted mandibular overdentures. Some studies reported that implant-retained overdentures restored with ILP presented lower survival rates compared to those restored with DLP [36, 37], but others found that both ILP and DLP groups showed similar MBL with successful osseointegration [34, 38]. Therefore, it is still unknown whether ILP could provide acceptable MBL for the unsplinted mandibular implant-retained overdentures in the clinic.

Consequently, we quantitatively compared the effects of immediate and delayed loading protocols on MBL around implants for unsplinted mandibular implant-retained overdentures in this systematic review and meta-analysis, to inform dental practitioners about the selection of appropriate loading protocol for the long-term clinical success of restorations.

## Methods

The present meta-analysis was performed in accordance with the Preferred Reporting Items for Systematic Reviews and Meta-Analyses (PRISMA) guidelines. The protocol was registered priori in the International Prospective Register of Systematic Reviews (PROSPERO) (www.crd.york.ac.uk/PROSPERO/) (registration number: CRD42020159124, Additional file 1, Details in Supplementary Information).

### PICOS question

According to the recommendations of the Centre for Evidence-Based Medicine (University of Oxford, Oxford, UK), the PICOS (participants, interventions, comparisons, outcomes, and study designs) question was as follows: *How is the effect on MBL around implants after ILP in the unsplinted mandibular implant-retained overdentures when compared to DLP?*

Participants: edentulous patients restored with unsplinted mandibular implant-retained overdentures.

Intervention: ILP for unsplinted mandibular implant-retained overdentures.

Comparisons: DLP for unsplinted mandibular implant-retained overdentures.

Outcomes: MBL with a minimum follow-up of one year.

Study designs: randomized controlled trials (RCTs), controlled clinical trials (CCTs), and cohort studies.

### Inclusion criteria

Based on the PICOS question, a study must fulfil the following inclusion criteria:

- Clinical studies on human subjects only.
- Studies with adults over 20 years of age who had mandibular edentulism restored by unsplinted implant-retained overdentures.
- Studies with a minimum follow-up of 1 year.
- Studies comparing the quantitative outcomes of MBL around implants in unsplinted mandibular implant-retained overdentures restored with ILP and DLP.
- Studies with details of measuring techniques.
- RCTs, CCTs, prospective and retrospective cohort studies.
- Studies reported in English language only.

### Exclusion criteria

In addition, if a study met any of the following exclusion criteria, it was excluded from the study:

- Case reports, review papers.
- Overdentures retained by a single implant only.
- Diameter of implants narrower than 3 mm (mini-implant).
- Duplicate studies based on the same patient cohorts.
- Studies with sample size less than ten.

### Information sources and literature search

The literature search was conducted independently by two independent assessors (W.L. and H.C.). Any disagreement was resolved by discussion between the two assessors. Three online electronic databases, including PubMed, EMBASE, and CENTRAL (Cochrane Library), were searched for relevant scientific reports published in the English language on December 02, 2020. No time filter was applied. The online search was conducted with the search strategy combining both the MeSH and free text words with high sensitivity and adaptation for the databases (Table 1). For the “grey” literature (e.g. unpublished and ongoing studies, conference abstracts, dissertation and thesis), the ClinicalTrials.gov, System for Information on Grey Literature in Europe (OpenGrey), National Technical Information Service (NTIS), and ProQuest Dissertation Abstracts, and Thesis databases were also searched. Furthermore, hand search was performed to identify the eligible reports based on the reference lists of related trials and reviews as a complement.

**Table 1:** Search strategy for the current systematic review

Step	Search strategy
#1	("overdenture"[MeSH Terms]) OR (implant overdenture) OR (IOD) OR (denture overlay)
#2	("Immediate Dental Implant Loading"[MeSH Terms]) OR (immediate loading) OR (delayed loading) OR (conventional loading)
#3	("Controlled Clinical Trial" [Publication Type]) OR (random*) OR (control*) OR (prospective) OR (retrospective)
#4	#1 AND #2 AND #3

### Study selection

After pooling the full search results from all database, literatures with repetitive contents were excluded. Two assessors (W.L. and H.C.) independently screened the titles and abstracts of studies, and irrelevant reports were discarded. Then the full-text evaluation of articles was carried out by the same two assessors to select reports that met all inclusion criteria as well as to exclude reports according to any of the exclusion criteria. Any disagreement about whether a study should be included was resolved by discussion or arbitrated by a third assessor (L.S.). In addition, the kappa statistic was used to measure agreement between the independent assessors.

### Data collection and data items

The data were extracted from included reports and cross-checked by two assessors (W.L. and H.C.) independently. If there was a discrepancy on the data extraction during this process, the third assessor (L.S.) was consulted and an agreement was finally reached through a consensus discussion. A data collection form was developed a priori to record the extracted information. The following data were included: study, study design, total number of patients, age, edentulous region, number of implants (per patient), implant system, implant diameter, implant length, torque of implants, attachment type, comparison, number of patients in ILP/DLP, loading time of ILP/DLP, radiographic method, marginal bone loss, dropout (patient).

During the data extraction, it was found that one of the studies reported the mean and the standard deviation of vertical bone loss at four different sites (i.e. distal, labial, mesial, and lingual), while the other studies measured MBL at distal and mesial sites around the inserted implants. Thus, only the average values of MBL at distal and mesial sites in these studies were included in the following meta-analysis.

### Risk of bias in individual trials

The risk of bias in the included RCTs were evaluated by the Cochrane Collaboration’s tool (RevMan v5.3) [39]. According to the bias indices, three different levels including low, moderate, and high were used to classify the risk of bias within RCTs [40]. The Newcastle–Ottawa scale (NOS), as an ordinal star-rating scale, was employed for the assessment of methodological quality of non-RCTs. In NOS, a higher score represented a higher report quality of cohort study [41]. The assessments were carried out by two independent assessors (W.L. and J.Z.). Any disagreements were discussed and resolved until consensus was reached.

### Summary measures and synthesis of results

Statistical analyses were performed via the RevMan (RevMan v5.3, Cochrane Collaboration) and Stata (Stata MP v14, StataCorp LP) software. To compare MBL of ILP group with DLP group, the weighted mean differences (WMDs) with 95% confidence intervals (95% CIs) for these continuous outcomes was calculated. The results were provided with a fixed-effect or random-effects model [42]. Statistical heterogeneity was measured by the Chi^2^ statistic and I^2^ statistic [40].

### Risk of bias across studies

When there are at least 10 studies included in the present meta-analysis, tests for funnel plot asymmetry was drawn. Otherwise, Egger’s test was employed to assess the publication bias [40, 43].

### Additional analyses

The subgroup analysis was carried out among different attachment types of unsplinted mandibular implant-retained overdentures including Locator attachments, ball anchors and magnetic attachments, in order to reveal whether the overall estimate effect would be influenced by different attachment types. Sensitivity analyses were performed by removing individual trials from the meta-analysis, to see whether the overall effect would be affected and thus to reveal the robustness of the results.

## Results

### Study selection

**Fig. 1:**
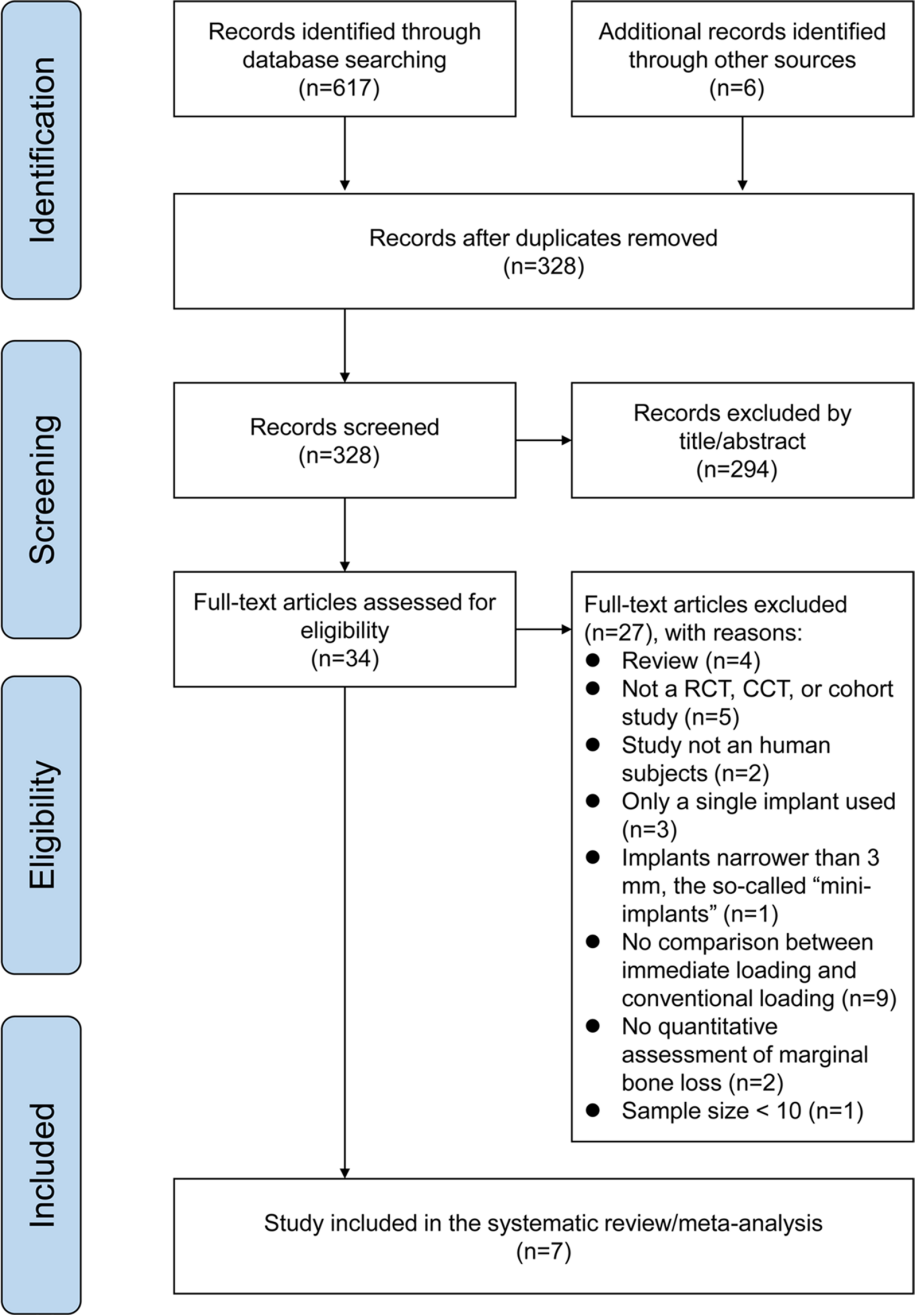
PRISMA flow program for study selection of this systematic review and meta-analysis process

Six hundred and seventeen and six records were identified through database search and hand search respectively. Through removal of repetitive records and initial screening by titles and abstracts, 34 records remained. After full-text review, 27 studies were excluded as they did not meet the eligibility criteria and a total of seven articles were included for this review and meta-analysis (Fig. 1).

The kappa value for the inter-investigator agreement of initial screening by titles and abstracts was 0.96 and that of full-text evaluation was 0.91, both presented an “almost perfect” inter-agreement [44].

### Study characteristics

Table 2 showed the characteristics of the included seven studies. All studies were published from 2007 to 2020, and 191 recruited participants with 400 implants were included all together. Among these seven studies, five (71%) were RCTs and two (29%) were prospective cohort studies. In six studies [45–50], overdentures in ILP groups were attached to the implants immediately after implantation, while in the other one study, the restorations in the ILP group were performed seven days after the surgery [51]. In DLP groups of all included studies [45–51], the healing time after implant surgery was no less than three months. Four studies [45–48] disclosed the specific ranges of inserting torque, and the other three studies [49–51] did not report the insertion torque. Moreover, Locator attachments were employed in three studies [45, 47, 48] and ball anchors were utilized in the other four literatures [46, 49–51]. In regard to “implants failed (at 12 months, implants)”, no implant failure was reported in ILP and DLP groups of three included studies [45, 46, 51]. Three studies [47–49] reported two implant failures occurring in ILP group and no implant failure in DLP group each. One study [50] revealed four implants failures in ILP group and two implant failures in DLP group. Thus, ILP group demonstrated a higher implant failure rate of 5.03% (10 of 199 implants), compared with 1.00% failure rate (2 of 201 implants) in DLP group.

**Table 2:** Characteristics of included studies

Nr	Study (year of publication)	Study design	Total number of patients	Age (year) Mean ± SD	Edentulous region	Number of implants (per patient)	Implant system	Implant diameter (mm)	Implant length (mm)	Torque of implants (N·cm)	Attachment type	Comparison	Number of patients in ILP/DLP	Loading time of ILP/DLP	Radiographic method	Marginal bone loss (mm) Mean ± SD	Implants failed (at 12 months, implants)
1	Kutkut et al. [45]	RCT	20	≥ 22	Mandible	2	BioHorizons	3.8	12	≥ 35	Locator attachment	Immediate loading	10	Immediately	CBCT	0.85 ± 0.69	0
												Delayed loading	10	3 months		1.33 ± 1.47	0
2	Aunmeungtong et al. [46]	RCT	40	ILP; 66.65 ± 6.28	Mandible	2	PW Plus	ILP; 3.5	ILP; 12	30–55	Ball anchor	Immediate loading	20	Immediately	Panoramic	0.53 ± 0.41	0
				DLP; 73.8 ± 10.4				DLP; 3.75	DLP; 10			Delayed loading	20	3 months		0.6 ± 0.45	0
3	Schincaglia et al. [47]	RCT	32	ILP; 66.6 ± 10.2	Mandible	2	Astra Tech	4	ILP; 8, 11, 13	≥ 20	Locator attachment	Immediate loading	15	Immediately	CBCT	0.25 ± 0.50	2
				DLP; 66.2 ± 8.6					DLP; 11, 13, 15			Delayed loading	15	3 months		0.54 ± 0.50	0
4	Elsyad et al. [48]	RCT	36	ILP; 60.3 ± 5.01	Mandible	2	Tiologic	3.7; 4.2; 4.8	11; 13; 15	≥ 35	Locator attachment	Immediate loading	16	Immediately	Standardized periapical radiograph	1.05 ± 0.18	2
				DLP; 58.9 ± 6.69								Delayed loading	17	3 months		0.87 ± 0.13	0
5	Elsyad et al. [49]	RCT	36	ILP; 63.2 ± 2.69	Mandible	2	Spectra system	3.7; 4.7; 5.7	10; 13; 16	NR	Ball anchor	Immediate loading	15	Immediately	CBCT	0.58 ± 0.53	2
				DLP; 64.6 ± 3.01								Delayed loading	15	3 months		0.55 ± 0.42	0
6	Turkyilmaz et al. [51]	Prospective	20	ILP; 62.3 ± 8	Mandible	2	TiUnite	3.75	15	NR	Ball anchor	Immediate loading	10	7 days	Standardized periapical radiograph	0.28 ± 0.26	0
				DLP; 63.2 ± 7								Delayed loading	10	3 months		0.27 ± 0.30	0
7	De Smet et al. [50]	Prospective	20	ILP; 58 (44–68)	Mandible	3	Nobel Bicon	3.75 to 4	≥ 13	NR	Ball anchor	Immediate loading	9	Immediately	Standardized periapical radiograph	1.07 ± 0.60	4
				DLP; 64 (33–72)								Delayed loading	9	3–4 months		0.47 ± 0.60	2

*Nr*, number; *ILP*, immediate loading protocol; *DLP*, delayed loading protocol; *RCT*, randomized controlled trial; *NR*, not reported; *CBCT*, cone beam computed tomography

### Risk of bias within studies

**Fig. 2:**
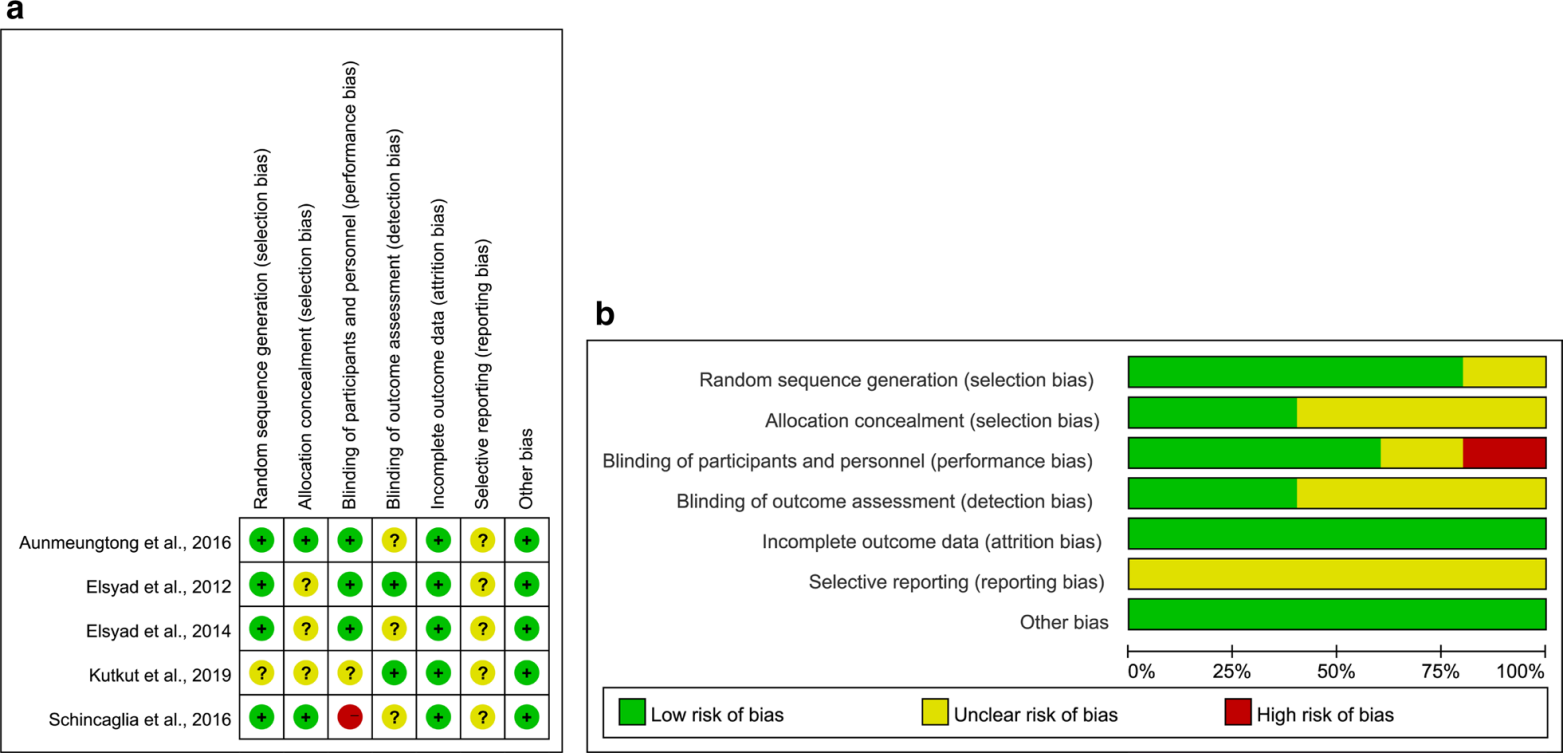
Quality assessment and risk of bias of the included RCTs assessed by review authors with the RevMan v.5.3 software: **a** risk of bias summary showing review authors' judgments about risk of bias items for each included RCT; **b** risk of bias graph showing review authors' judgments about each item's risk of bias presented as percentages for all included RCTs

Figure 2 illustrated the quality assessments and risk of bias of the included RCTs. Four RCTs [45, 46, 48, 49] were of unclear risk of bias while one study [47] included a high risk of bias. Two RCTs had a low risk of selection bias and three RCTs did not provide detailed information in terms of random sequence generation or allocation concealment. Only one RCT [47] (20%) reported a high risk of performance bias as only one experienced operator performed all the surgeries in ILP group and DLP group. Three studies [46–48], which did not provide enough information about the binding of outcome assessment, had an unclear risk of detection bias. All studies reported low risk of attrition bias with no missing data [45, 46, 49] or reasonable explanations for only one missing data [47, 48]. The reporting bias was classified at an unclear level in 80% of the trials [45, 46, 48, 49], for insufficient information was available to judge the risk level. These studies appeared to be free of other sources of bias. Table 3 presented the quality assessment results of two prospective cohort studies [50, 51]. The overall risk of bias was deemed to be low (seven stars) in the two cohort studies, and these studies were proved to be of an acceptable quality [41, 52, 53]. As the two studies did not report the derivation of the cohorts, it was unclear to judge the representativeness of their exposed cohorts. Though two subjects of one study [50] lost to follow up, the description of those lost was provided in detail and then the adequacy of follow up was considered with low risk of bias.

**Table 3:** Quality assessment and risk of bias of the included non-randomized studies

Study	Coding manual for cohort studies	Newcastle–Ottawa Scale
Turkyilmaz et al. [51]	Selection	
	(1) Representativeness of the exposed cohort	d
	(2) Selection of the non-exposed cohort	a☆
	(3) Ascertainment of exposure	a☆
	(4) Demonstration that outcome of interest was not present at start of study	a☆
	Comparability	
	(1) Comparability of cohorts on the basis of the design or analysis	a☆
	Outcome	
	(1) Assessment of outcome	a☆
	(2) Was follow-up long enough for outcomes to occur	a☆
	(3) Adequacy of follow up of cohorts	a☆
	Total Scale	☆☆☆☆☆☆☆
De Smet et al. [50]	Selection	
	(1) Representativeness of the exposed cohort	d
	(2) Selection of the non-exposed cohort	a☆
	(3) Ascertainment of exposure	a☆
	(4) Demonstration that outcome of interest was not present at start of study	a☆
	Comparability	
	(1) Comparability of cohorts on the basis of the design or analysis	a☆
	Outcome	
	(1) Assessment of outcome	a☆
	(2) Was follow-up long enough for outcomes to occur	a☆
	(3) Adequacy of follow up of cohorts	b☆
	Total Scale	☆☆☆☆☆☆☆

Selection: (1) d: no description of the derivation of the cohort; (2) a: drawn from the same community as the exposed cohort ☆; (3) a: secure record (e.g., surgical records) ☆; (4) a: yes ☆. Comparability: (1) a: study controls for ____ (select the most important factor) ☆. Outcome: (1) a: independent blind assessment ☆; (2) a: yes (select an adequate follow up period for outcome of interest) ☆; (3) a: complete follow up—all subjects accounted for ☆; b) subjects lost to follow up unlikely to introduce bias—small number lost → ____ % (select an adequate %) follow up, or description provided of those lost) ☆

### Results of individual studies and synthesis of data

**Fig. 3:**
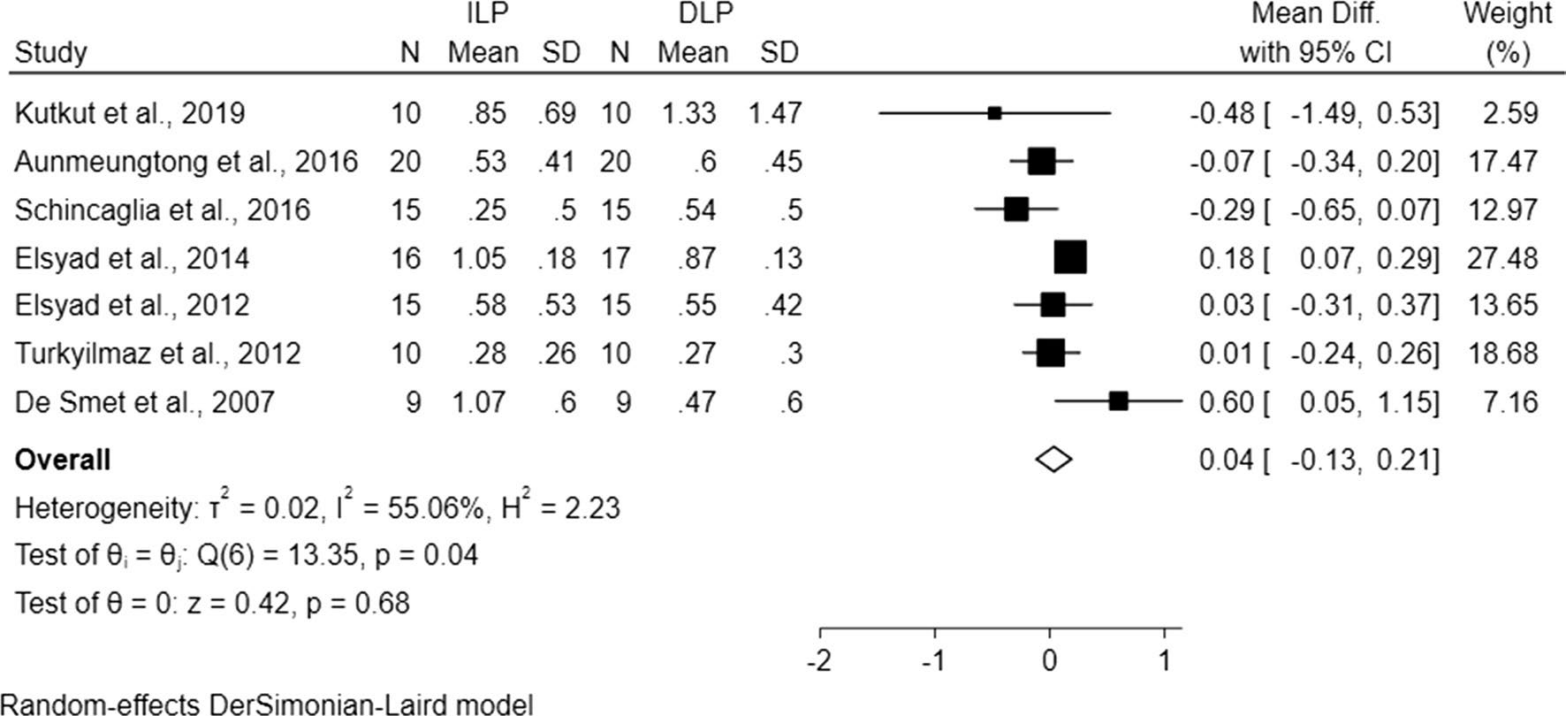
Forest plot generated by Stata MP v.14 software for comparison of immediate loading protocol versus delayed loading protocol with regard to marginal bone loss. ILP, immediate loading protocol; DLP, delayed loading protocol

In this review, 191 patients with a follow-up of no less than 12 months were pooled for the synthesis of data and the meta-analysis result of seven studies was illustrated with the forest plot (Fig. 3). As a result of the comparison of MBL between ILP and DLP groups, a substantial heterogeneity [39] (*P* = 0.04, *I*^*2*^ = 55.06% > 50%) was found. Therefore, instead of the fixed-effect model, DerSimonian–Laird model as a random-effect model was applied according to the STATA technical bulletin and Cochrane handbook [40, 54]. No statistically significant difference of MBL was detected (WMD 0.04, CI − 0.13 to 0.21, *P* = 0.68) between ILP and DLP group.

### Risk of bias across studies

Because the limited number of included studies (n < 10), the publication bias assessment was conducted with Egger’s test in the current meta-analysis. The result of Egger’s test indicated that no significant bias could be found among seven included studies (*P* > 0.05). Nevertheless, this evaluation of risk of publication bias should be considered as a reference only owing to the limited quantity of articles.

### Additional analysis

**Fig. 4:**
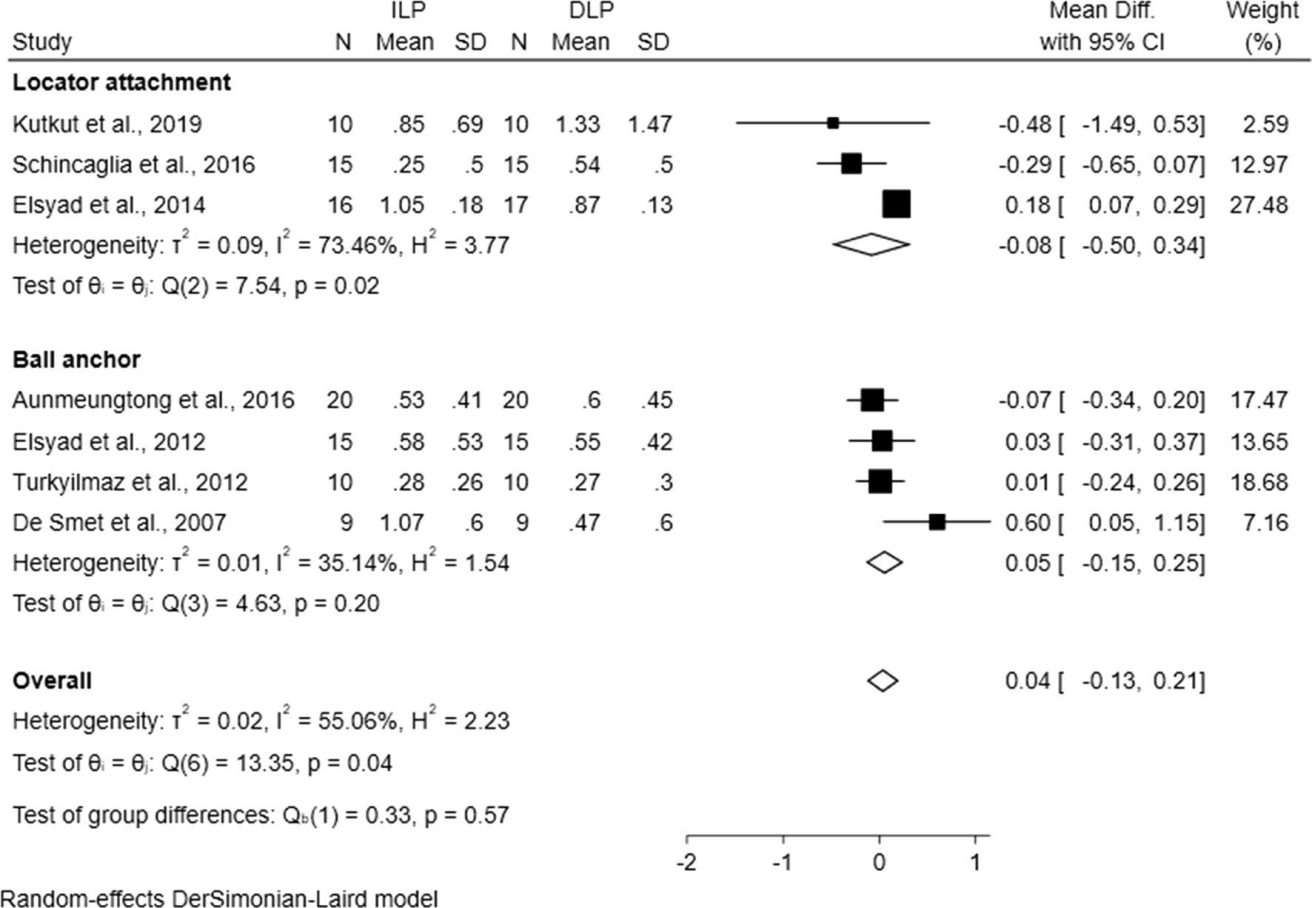
Forest plot generated by Stata MP v.14 software for subgroup comparison of immediate and delayed loading protocols with regard to marginal bone loss in Locator attachments subgroup and ball anchors subgroup. ILP, immediate loading protocol; DLP, delayed loading protocol

**Fig. 5:**
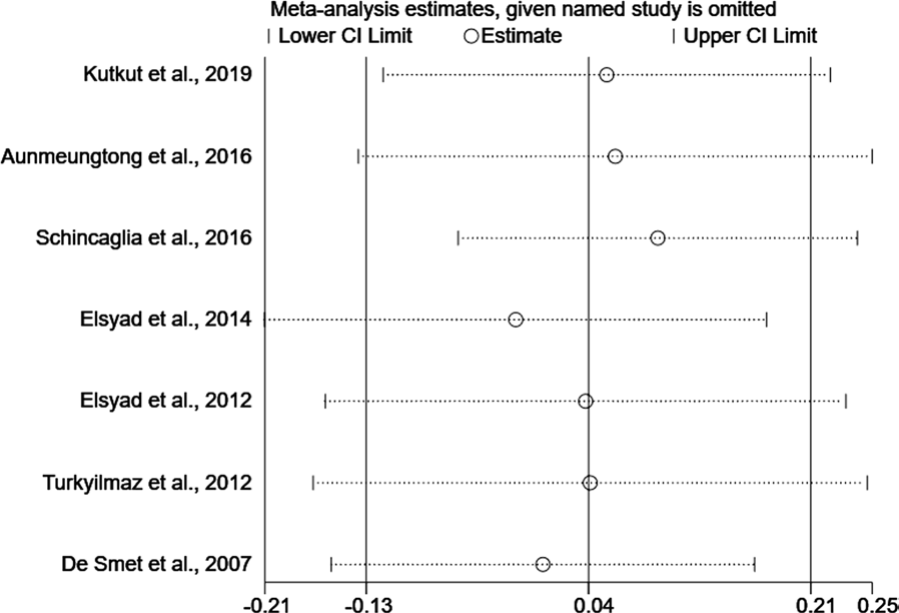
Sensitivity analyses generated by Stata MP v.14 software for comparison between immediate and delayed loading protocols with regard to marginal bone loss

According to the different attachments applied in these studies, two subgroups (i.e. Locator attachment vs. ball anchor) were set. Figure 4 showed the comparison of average MBL in the subgroups respectively. In either the Locator attachments (three trials, WMD − 0.08, CI − 0.50 to 0.34, *P* > 0.05) or the ball anchors (four trials, WMD 0.05, CI − 0.15 to 0.25, *P* > 0.05) subgroup, no significant difference was detected between ILP and DLP. In the sensitivity analyses, one single study was deleted from the overall pooled analysis each time and it was found that the exclusion of individual studies did not show any influence on the overall MBL (Fig. 5). Moreover, with the omission of two prospective studies, there was still no significant difference between ILP and DLP groups (WMD − 0.02, CI − 0.23 to 0.19, *P* > 0.05).

## Discussion

The present systematic review was based on the results from five RCTs and two non-RCTs with a total of 191 patients. The meta-analysis was conducted to compare the MBL around inserted implants of unsplinted mandibular overdentures between ILP and DLP groups, and the result did not reveal any significant difference (*P* > 0.05). Further, the subgroup analysis showed similar results about the MBL between the two different loading protocols in either the Locator attachments or the ball anchors subgroup (*P* > 0.05), suggesting that different attachment types employed in the included studies did not result in any difference on the result of meta-analysis, and might not be the source of cross-study heterogeneity. Additionally, in both ILP and DLP groups of these included studies, the average MBL surrounding implants of unsplinted implant-retained overdentures were lower than 2 mm, indicating that the results were all clinically acceptable as far as MBL was concerned [32].

Several studies reported that the initial healing of implants with immediately loaded mandibular overdentures might be impaired by the resultant restoration movement, immediate abutment connection, and early contact with oral microbial plaque [55–57]. However, the present meta-analysis reported no detectable difference in the MBL around immediately loaded implants compared to the DLP group, which is also in line with the evidence from some previous systematic reviews [34, 58]. This may be attributed to the early mechanical strain and a lack of the second-stage surgery in ILP [59–61]. Mechanical loading from the overdentures in ILP might act as a stimulator to alveolar bone formation and then lead to high bone fractions [61]. The early mechanical strain in the bone-to-implant contact surface was found to have a positive effect on the initial phase of bone healing [59, 60]. Moreover, second-stage surgical operations could bring additional trauma and damage tissues, leading to marginal bone loss [62, 63]. DLP with a second-stage surgery might be accompanied by the loss of underlying bone around implants compared with ILP [64]. Noticeably, Sanz-Sanchez et al. [25] and Kern et al. [65] synthesised both the fixed implant-supported and removal implant-retained restorations and reported that the ILP group presented an even less MBL compared to DLP group. This result may due to the combined discussion of fixed implant-supported and removal implant-retained restorations. The early mechanical strain from fixed dentures is usually much higher than that from removable restorations, thus the positive effect of immediate loading on MBL [61] is supposed to be more obvious.

Besides different loading protocols, marginal bone level could also be influenced by some factors such as implant primary stability and alveolar bone condition. In seven included studies, the effect of these factors on the MBL has been carefully controlled. The insertion torque value and implant stability quotient (ISQ) in these studies revealed clinically acceptable micromotion of implants [18, 66], and all implants sites of included cases were the alveolar bone of anterior mandible which was considered with the highest bone density compared to the alveolar bone of other oral regions [67]. Therefore, the primary stability, as an essential prerequisite for implant success, met the implant loading requirement for both ILP and DLP.

In this review, the early loading protocol, in which implants were put in function between one week and two months after placement [23], was not discussed, for this loading protocol could not reduce healing time significantly compared with ILP, whereas would increase the period with low masticate efficiency, and is seldom applied on the implant-retained overdentures in the clinic.

Apart from MBL around implants, the dropouts of patients as well as the implant failures were reported in these included studies. Patient dropouts commonly occur in long-term studies, due to death, illness, leaving the city, or many other reasons, it was considered as a quality index of RCT rather than a parameter of clinical outcomes, while the implant failure is commonly recognized as a key index for prognosis of implant treatment [68]. Therefore, in the current meta-analysis, only the data of implant failure was extracted from the included studies. MBL is triggered by multiple factors, such as gender, age, bone density, and type of connection. And the definition of implant failure requires the assessment of these features of each implant and patient [69]. Hence, given the crucial role of MBL in the prognosis of long-term implant failure, all possible efforts should be employed to decrease the MBL around implants and build a strict follow-up maintenance program. In this meta-analysis, the MBL at the first year of loading was chosen as the main outcome and the early implant failures of included studies occurring in the first 12 months were also reported (Table 2). Since three included studies [49–51] here did not report specific values of inserted torque in the implant surgery which had an obvious impact on the early survival rate of implants [70], a robust conclusion about implant success could not be drawn in this review. Therefore, this index was not taken into consideration in this present meta-analysis.

MBL was employed as the only criterion to evaluate the therapeutic effects of immediate and delayed loading protocols in this study. However, this parameter indeed has its drawback. The raw MBL data at 1-year post-loading is a static index and it could only present the status of peri-implant tissue at one single time point, thus the reliability of MBL is controversial. Accordingly, the rate of MBL as a new index was proposed by Galindo-Moreno et al. recently [69]. As remodelling of marginal bone is a dynamic process, the rate of MBL is calculated in millimetre/month (mm/m) and could change over time [69, 71]. Galindo-Moreno et al. [69] found that the progression of MBL tends to be higher and the risk of implant failure could be significantly increased when the rates of MBL was higher than 0.44 mm at six months post-loading. The new index may better help dentists predict future bone changes in the early stage and then establish a strict maintenance recall for patients. Meanwhile, a more definite evaluation of clinical outcomes between these two different loading protocols could be carried out in a short observation time rather than a minimum follow-up of one year. Thus, the rate of MBL might be a more suitable criterion for implant success in the clinic.

This was the first study to systemically evaluate the MBL of immediate loading compared to delayed loading in unsplinted mandibular implant-retained overdentures. The protocol of this study was registered in PROSPERO in advance and performed strictly in accordance with PRISMA guidelines. Apart from online search, manual search was performed based on the references of selected studies and related reviews, in order to discover qualified trials which might not be included in the databases. Therefore, compared with individual studies, it is a more convincing clinical suggestion of loading protocol selection of unspinted mandibular implant-retained overdentures to practitioners. However, there are some limitations of this study. The implant number and implant system in the included studies had not achieved complete consistency, which might cause the considerable heterogeneity in the meta-analysis. Additionally, only seven clinical trials were included and two of them were prospective cohort studies. Due to the limited number of the trials, the results of this study might lack sufficient evidence. Moreover, although the result of meta-analysis after removing two included prospective studies [50, 51] remained the same, the validity of analysis might also be slightly compromised since no randomization of participant allocation could be employed in cohort studies. Thus, further high-quality, well-designed RCTs with large sample size are required to appraise the efficacy of different loading protocols on MBL around implants in unsplinted mandibular implant-retained overdentures.

## Conclusions

Based on the results of this systematic review and meta-analysis, the MBL around implants restored with ILP showed no significant difference with that of implants restored with DLP for unsplinted mandibular implant-retained overdentures. The subgroup analysis suggested that either the Locator attachments or the ball anchors employed in the included studies would not result in any difference on the result of meta-analysis. However, considerable heterogeneity was observed across the included studies. Also, the limited number of trails and no randomization of participant allocation in the included cohort studies might compromise the validity of analysis. Further high-quality RCTs with robust study design and large sample size are needed to strengthen the evidence base and identify the effect of immediate and delayed loading protocols on MBL around implants in unsplinted mandibular implant-retained overdentures.

## Supplementary Information

**Additional file 1**. The PROSPERO protocol of this systematic review.

## Data Availability

We declared that the datasets used and/or analyzed in the manuscript, including all relevant raw data, are available from the corresponding author on reasonable request.

## Abbreviations

MBL: Marginal bone loss
RCTs: Randomized controlled trials
CCTs: Controlled clinical trials
ILP: Immediate loading protocol
DLP: Delayed loading protocol
WMDs: Weighted mean differences
CIs: Confidence intervals
PRISMA: Preferred Reporting Items for Systematic Reviews and Meta-Analyses
PROSPERO: International Prospective Register of Systematic Reviews
PICOS: Participants, interventions, comparisons, outcomes, and study designs
NOS: Newcastle–Ottawa scale
ISQ: Implant stability quotient
